# ElasticGS: Pose-Aware Dynamic Gaussian Adaptation for Geometry-Consistent Human Digital Twin Reconstruction from Monocular Video

**DOI:** 10.3390/s26217014

**Published:** 2026-11-06

**Authors:** Dong Zhou, Zhe Huang, Haiyang Li, Mengyun Cao, Chih-Cheng Chen

**Affiliations:** 1 School of Computer Engineering, Jimei University, Xiamen 361021, China; 202411835014@jmu.edu.cn (D.Z.); 202511835009@jmu.edu.cn (Z.H.);; 2 Department of Automatic Control Engineering, Feng Chia University, Taichung 40724, Taiwan

**Keywords:** 3D Gaussian Splatting, vision-based 3D sensing, human digital twin, dynamic human reconstruction, surface integrity evaluation

## Abstract

Vision-based 3D sensing provides an effective approach for constructing human-centric digital twins in applications such as ergonomic assessment and human–robot collaboration. However, reconstructing reliable dynamic human digital twins from visual sensor observations remains challenging due to large articulated deformation, which often causes surface discontinuities in existing 3D Gaussian Splatting (3DGS)-based human avatars. Although current methods achieve high image-level rendering quality, their Gaussian primitives are not explicitly adapted to pose-induced surface deformation, resulting in cracks and holes during motion. In this work, we propose ElasticGS, a geometry-consistent human digital twin reconstruction framework for vision-based 3D sensing. ElasticGS introduces a training-free Pose-Aware Dynamic Covariance Adaptation strategy, which dynamically adjusts the rotation and anisotropic scale of Gaussian primitives according to local human surface deformation. Specifically, localized Principal Component Analysis (PCA) is performed on deformed SMPL-X mesh neighborhoods to estimate surface stretching directions and magnitudes, enabling Gaussian representations to maintain continuous surface coverage under complex poses. Furthermore, we propose the Avatar Surface Integrity Score (ASIS), a reference-free metric that evaluates structural completeness and surface compactness of reconstructed human avatars without requiring pixel-aligned ground truth. Experiments on the X-Humans, AvatarReX, and People-Snapshot datasets demonstrate that ElasticGS significantly improves surface integrity while maintaining competitive image-level sensing consistency. Compared with ExAvatar, ElasticGS maintains satisfactory rendering quality while improving ASIS from 0.741 to 0.961 on X-Humans, from 0.871 to 0.949 on AvatarReX, and from 0.874 to 0.952 on People-Snapshot. These results demonstrate the effectiveness of pose-aware covariance adaptation for robust human digital twin reconstruction in vision-based 3D sensing systems.

## 1. Introduction

Vision-based 3D sensing is increasingly used to perceive, reconstruct, and monitor human activities in cyber–physical environments. In ergonomic assessment and human–robot collaboration visualization, RGB cameras, RGB-D sensors, LiDAR, and multi-view imaging systems provide continuous observations for building dynamic human digital representations. However, practical sensing systems often operate under imperfect observation conditions. Monocular cameras suffer from depth ambiguity, RGB-D sensors may produce noisy or missing depth values, LiDAR provides relatively sparse surface samples for detailed human modeling, and multi-view systems are often affected by limited camera placement, inter-view occlusion, and self-occlusion. As a result, human surfaces reconstructed from real-world observations are frequently incomplete, unevenly sampled, or temporally inconsistent, particularly around limbs, joints, and clothing regions. Compared with static object reconstruction, human-centered 3D sensing is more challenging because the target surface undergoes large articulated and non-rigid deformation while the observations remain sparse or partially occluded. Therefore, an effective sensing-oriented reconstruction system should preserve not only photorealistic appearance but also geometrically continuous and structurally complete body surfaces under novel poses.

Recent neural rendering techniques, including Neural Radiance Fields (NeRF) [1] and 3D Gaussian Splatting (3DGS) [2], have significantly advanced dynamic human reconstruction from visual sensor data. In particular, 3DGS represents a scene using explicit Gaussian primitives and supports efficient differentiable rasterization, making it attractive for real-time human digital twin applications. When combined with parametric human models such as SMPL-X [3], 3DGS-based methods can reconstruct animatable human avatars from monocular or multi-view videos and animate them with novel poses. This capability is valuable for vision-based 3D sensing systems because it enables compact, renderable, and controllable representations of human subjects from sensor-captured imagery.

**Figure 1:**
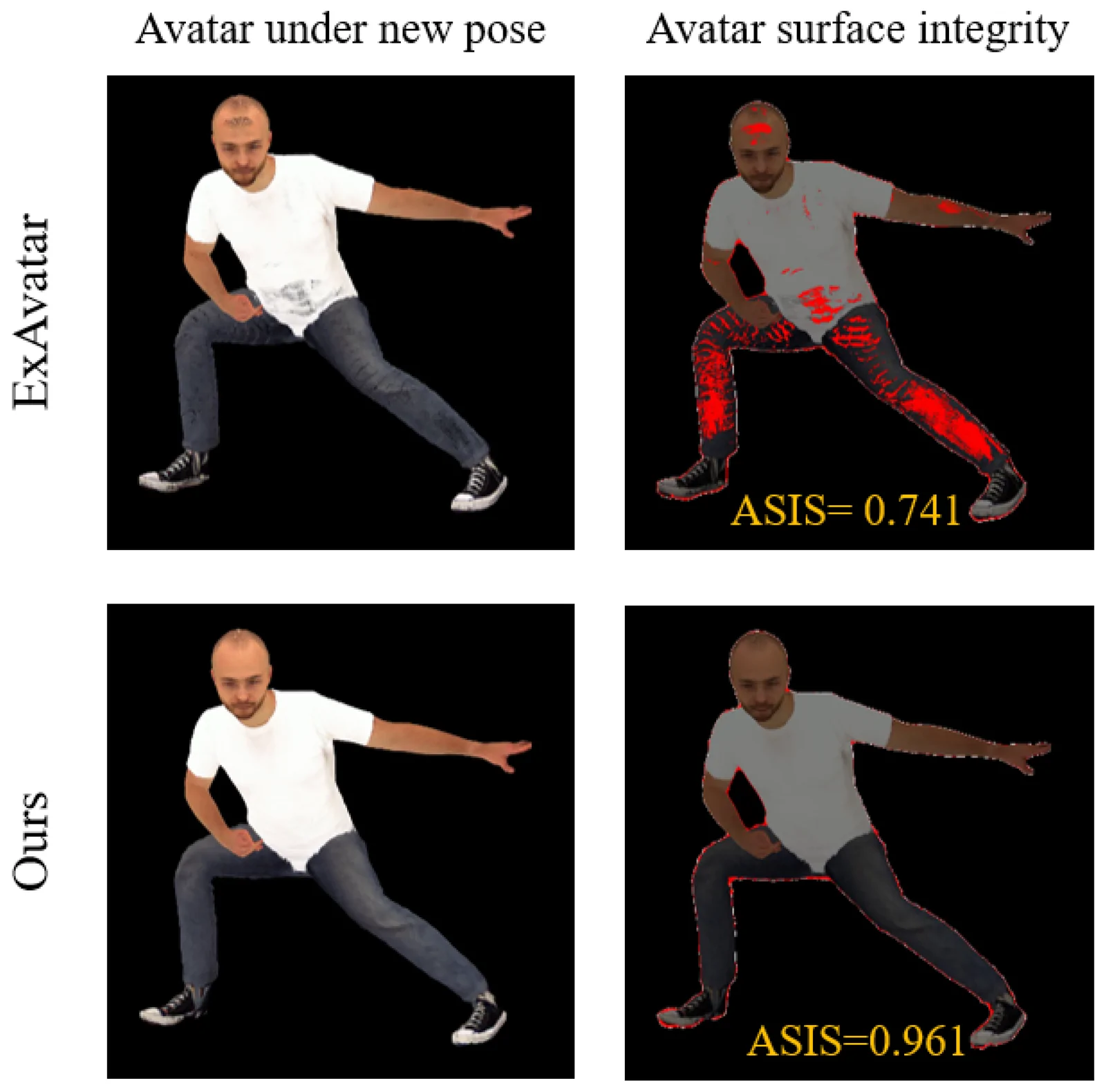
Comparison between our reconstructed avatar and ExAvatar under novel poses. The selected object is 00034 from X-Humans. (**Left**) The rendered image of the avatar performing the novel motion. (**Right**) The visualization and quantitative results of the avatar surface integrity.

However, current 3DGS-based human reconstruction methods still face a critical limitation when used in sensing scenarios involving large articulated motion. Most methods primarily optimize Gaussian positions, colors, and opacities for image-level fidelity, while the covariance support of Gaussian primitives is either isotropic or learned in a static canonical pose [4,5,6,7,8]. As a result, when the underlying human surface is stretched or compressed during motion, the Gaussian primitives cannot adapt their spatial coverage accordingly. As shown in Figure 1, this mismatch produces visible surface cracks and density holes, which may be acceptable in some appearance-oriented rendering applications but are problematic for 3D sensing applications that require reliable surface continuity for monitoring, measurement, and downstream analysis.

To address this issue, we propose ElasticGS, a geometry-consistent dynamic human reconstruction framework for vision-based 3D sensing. Instead of treating Gaussian primitives as rigid particles attached to a moving body surface, ElasticGS models them as locally adaptive surface elements whose covariance is updated according to pose-induced mesh deformation. Specifically, during motion-driven inference, we perform localized PCA on the deformed SMPL-X mesh neighborhood of each Gaussian primitive and use the resulting principal directions and deformation magnitudes to analytically update its rotation and anisotropic scale. This training-free mechanism allows the Gaussian field to maintain more continuous surface coverage under large articulated motions.

Furthermore, existing evaluation protocols for dynamic human reconstruction predominantly rely on image-level fidelity metrics, such as PSNR, SSIM, and LPIPS, or reference-based geometric metrics, such as depth error, Chamfer Distance, and silhouette overlap. These metrics are useful for measuring appearance similarity or global geometric alignment, but they are not well suited for quantifying local density holes and surface discontinuities in pose-driven 3DGS avatars, especially when pixel-aligned ground truth images or complete reference meshes are unavailable. Although recent no-reference quality assessment methods have been explored for NeRF/NVS, 3DGS rendering, and point-cloud quality, they mainly target perceptual quality or generic reconstruction artifacts rather than the surface compactness of Animatable Gaussian human avatars [9,10,11,12]. To complement these metrics, we introduce the Avatar Surface Integrity Score (ASIS), a reference-free proxy-rendering metric that evaluates the structural completeness of 3DGS avatars by comparing an ideal morphological body mask with an effective density mask derived from Gaussian opacity accumulation.

The main contributions of this paper are as follows:

- We propose ElasticGS, a geometry-consistent dynamic human reconstruction framework for vision-based 3D sensing and human digital twin applications. The framework improves the structural reliability of 3DGS-based human representations under large articulated motions.
- We introduce a Pose-Aware Dynamic Covariance Adaptation method to address the issue of surface cracks in human avatars. This training-free approach explicitly couples local mesh geometric deformations with the covariance attributes of Gaussian primitives, enabling adaptive rotation and scaling in response to skin stretching.
- We propose the Avatar Surface Integrity Score (ASIS), a task-specific reference-free proxy metric for evaluating surface compactness and density continuity in 3DGS-based human avatars. Unlike conventional image-level metrics or reference-based geometric metrics, ASIS uses proxy alpha rendering and morphological mask construction to quantify motion-induced cracks and density holes when pixel-aligned ground truth or complete reference geometry is unavailable.
- We conduct experiments on X-Humans [13], AvatarReX [14] and People-Snapshot [15] to evaluate image-level consistency and geometric reliability. The results demonstrate that ElasticGS significantly reduces motion-induced cracks and density holes while maintaining competitive image-level reconstruction quality.

## 2. Related Work

### 2.1. Sensor-Driven Dynamic Human Reconstruction

Sensor-driven human reconstruction has been an active topic in the Sensors community, especially with the availability of RGB cameras, RGB-D sensors, and low-cost depth cameras for human modeling and digital-twin applications. Early work showed that personalized parametric human models can be incrementally built from a single RGB camera by exploiting motion cues [16]. RGB-D-sensor-based avatar reconstruction further demonstrated efficient full-body scanning and 3D avatar generation in indoor environments [17]. Beyond static scanning, dynamic non-rigid reconstruction with a single RGB-D sensor has been studied to recover complete and temporally consistent deformable models from sequential observations [18]. More recently, human-centric digital twins in industry have been reviewed as an important direction integrating RGB-D sensing, computer vision, pose estimation, and digital human modeling for industrial monitoring, ergonomic analysis, and human–robot collaboration support [19]. These studies indicate a progression from sparse pose or scan-based human sensing toward dense, dynamic, and geometry-aware human representations.

Recent advances in close-range remote sensing, photogrammetry, and multi-sensor 3D modeling have further enabled accurate reconstruction of dynamic scenes and human digital twins. Within this context, human-centered reconstruction has attracted growing attention, especially for applications requiring articulated motion capture, geometry-aware monitoring, and dynamic scene understanding. Early methods such as SCANimate [20] and CanonicalFusion [21] established the basis for learning deformable human templates from video observations through weak supervision and canonical-space modeling.

With the introduction of 3D Gaussian Splatting, several works have explored explicit Gaussian representations for dynamic human reconstruction. HUGS [22] combines SMPL priors with joint scene optimization to separate humans from static environments and achieve real-time motion-driven rendering from monocular videos. GauHuman [23] further improves reconstruction quality under complex poses through alternating optimization of skeletal parameters and Gaussian attributes. GART [24] introduces an articulated Gaussian template with explicit structural priors, reducing motion artifacts in monocular human reconstruction. Deblur-Avatar [25] addresses motion blur in dynamic sequences, while SplattingAvatar [26] demonstrates efficient surface-embedded Gaussian rendering on mobile devices. ExAvatar [4] extends this line by anchoring 3D Gaussians to the SMPL-X surface and reconstructing expressive whole-body avatars with detailed face and hand motion.

Despite these advances, existing sensor-driven human reconstruction methods still face a critical limitation under large articulated motion. In monocular or sparsely observed settings, self-occlusion, depth ambiguity, and strong non-rigid deformation make it difficult to preserve geometric consistency on the body surface. As a result, even visually plausible reconstructions may exhibit local cracks or density holes in stretched regions, which limits their reliability for digital twin analysis and geometry-aware downstream applications.

### 2.2. Gaussian-Based Surface Modeling for Vision-Sensor Reconstruction

Pose-guided deformation is a central problem in 3DGS-based dynamic human modeling because Gaussian primitives must be transformed from canonical space to posed space while preserving both appearance fidelity and geometric continuity. Existing methods generally follow several technical paradigms.

The first line of work relies on skeletal or template-driven deformation. HUGS [22] and Human Gaussian Splatting [27] define Gaussian primitives in a canonical human space and animate them using skinning-based transformations, where coarse articulated motion is further refined by local non-rigid deformation. GART [24] introduces an articulated Gaussian template to impose structural motion priors, while TAGA [28] extends Gaussian-based articulated modeling to template-free settings by correcting ambiguous skinning and enforcing cycle consistency. These approaches provide efficient pose control, but their deformation mechanisms mainly focus on Gaussian positions or skinning correspondences rather than explicitly adapting Gaussian covariance support to local surface stretching.

A second line of work embeds or parameterizes Gaussian primitives on explicit surfaces. SplattingAvatar [26] defines Gaussians through barycentric coordinates and surface displacements on triangle meshes, allowing the underlying mesh deformation to control Gaussian translation and rotation. ExAvatar [4] further anchors 3D Gaussians to the SMPL-X surface to reconstruct expressive whole-body avatars with facial and hand detail. Recent methods such as MonoCloth [29] and RMAvatar [30] improve monocular Gaussian avatars through clothing dynamics and rectified mesh-embedded deformation, while GAF [31], GASP [32], and D3GA [33] explore more specialized reconstruction settings. ASH [34] parameterizes animatable 3D Gaussians on a deformable clothed template and predicts motion-conditioned Gaussian attributes in texture space, thereby improving high-resolution rendering efficiency. Related surface-embedded Gaussian formulations have also been explored in head avatar modeling, such as FlashAvatar [35] and MeGA [36]. Although these methods improve animation stability and rendering efficiency, they do not necessarily ensure that the anisotropic support of each Gaussian primitive changes consistently with local surface expansion and compression.

A third line of work learns pose- or expression-conditioned Gaussian deformation through neural networks or basis representations. 3DGS-Avatar [8] learns a non-rigid deformation network for animatable clothed avatars, while Animatable Gaussians [37] and AniGaussian [7] model pose-dependent Gaussian attributes for high-fidelity human animation. For head avatars, Gaussian Head Avatar [38] and MonoGaussianAvatar [39] introduce learned deformation fields to capture complex expressions from sparse observations. In addition, 3D Gaussian Blendshapes [40] and RGBAvatar [41] extend classical blendshape modeling to Gaussian attributes, enabling efficient expression-driven animation. More recently, SeqAvatar [42] incorporates hierarchical motion context to better model non-rigid appearance variations. While these methods substantially enhance expressive capacity, their deformation is often learned implicitly from data and may generalize less robustly to unseen large-amplitude motions, especially when strong local stretching leads to sparse Gaussian coverage and structural discontinuities.

Recent methods such as 3DGS-Avatar, AniGaussian, ASH, and SeqAvatar learn pose-conditioned Gaussian deformation, non-rigid offsets, or motion-dependent Gaussian attributes. These methods improve animation quality and may implicitly reduce surface discontinuities by learning deformation priors from data. However, their deformation behavior is usually learned from the training distribution and may generalize less robustly to unseen large articulated motions. Different from these implicit deformation strategies, ElasticGS explicitly updates Gaussian covariance according to local surface stretching estimated from SMPL-X neighborhoods, providing a training-free and geometry-driven mechanism for improving surface coverage.

### 2.3. Evaluation Metrics for Sensor-Based Dynamic Human Reconstruction

In the field of dynamic human reconstruction and animation, establishing a multi-dimensional evaluation system is crucial for validating algorithm effectiveness. Existing metrics are primarily categorized into image-level quality, specific artifact detection, and geometric consistency. Mainstream 3DGS works (e.g., HUGS [22], GauHuman [23], GaussianAvatar [43], Animatable Gaussians [37], GaussianAvatars [44], and GPS-Gaussian [45]) widely employ image quality metrics like PSNR, SSIM, and LPIPS to evaluate novel view synthesis quality. As for specific artifact detection, FLIP [46] visualizes perceptual differences between rendered and reference images, whereas Depth L1 Error [47] and RMSE [48] reflect the continuity of geometric surfaces by calculating depth map deviations. However, these metrics based on 2D projections focus mainly on whether the visible area of the avatar is the same as that in the reference image. Regarding geometric structure, Mask IoU [49] and Boundary IoU [50] detect limb loss or background penetration by evaluating silhouette coverage, where Chamfer distance measures discrepancies between sampled reconstructed and reference surfaces [51] but does not directly isolate local coverage gaps. Normal consistency does not indicate whether Gaussian primitives provide complete surface coverage.

Nevertheless, the aforementioned metrics have significant limitations in practical applications, as they rely almost entirely on strictly aligned ground truth (GT) data. In tasks involving novel motion driving, pixel-aligned reference images are often unavailable, rendering GT-based difference evaluation ineffective. Simultaneously, conventional image metrics lack sensitivity to “density holes” caused by 3D spatial stretching. Therefore, there is an urgent need to construct a reference-free geometric continuity evaluation metric to quantitatively measure the degree of cracks and holes on the avatar surface.

## 3. Materials and Methods

### 3.1. Overview

This study aims to address the issue of surface “cracks” appearing in human avatars reconstructed by existing 3D Gaussian Splatting (3DGS) methods. Drawing inspiration from ExAvatar, we adopt the “hybrid mesh-Gaussian representation”, which leverages the topological structure of SMPL-X to explicitly guide the distribution of 3D Gaussian primitives. However, when handling novel poses, the geometric attributes (e.g., scaling and rotation) of ExAvatar’s Gaussian primitives are restricted to be isotropic, lacking physical geometric coupling with the stretching deformation of the mesh surface. This “inelastic” rendering characteristic leads to noticeable cracks when the underlying mesh undergoes obvious deformation (such as knee bending or arm raising), as Gaussian primitives cannot stretch accordingly to cover the increased surface area. This inelastic behavior may also introduce visual artifacts when Gaussian primitives fail to contract as the local surface area decreases.

**Figure 2:**
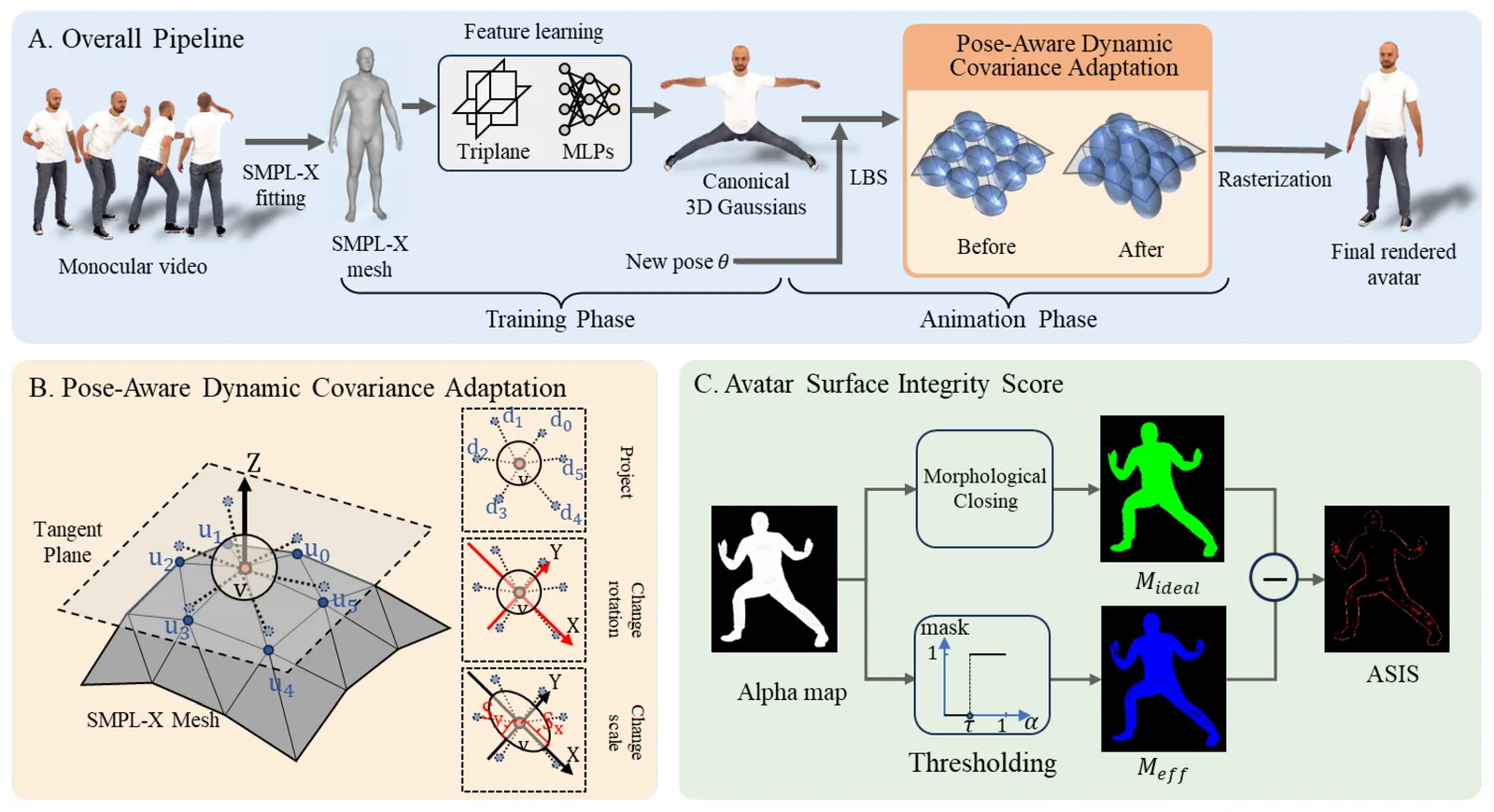
Overview of ElasticGS. (**Top**) The overall pipeline builds upon ExAvatar but introduces a training-free Pose-Aware Dynamic Covariance Adaptation method during the animation phase to eliminate surface cracks. (**Bottom Left**) Detail of the adaptation module: we perform PCA on the tangent plane of the deformed mesh to align and scale Gaussian primitives (visualized as ellipsoids) along the principal stretching direction ($e_{1}$). (**Bottom Right**) Illustration of the proposed ASIS metric, which quantifies surface integrity by comparing the ideal morphological mask ($M_{ideal}$) with the effective density mask ($M_{eff}$) derived from a proxy rendering pass.

As shown in Figure 2, our workflow consists primarily of two stages:

- Avatar Reconstruction(training phase). In this phase, we follow the training strategy of ExAvatar to learn the avatar representation in the canonical space from a monocular video. Specifically, we utilize a triplane feature field and Multi-Layer Perceptrons (MLPs) to regress the 3D Gaussian attributes (position, color, opacity, etc.) associated with each SMPL-X vertex in the canonical space. By minimizing the image reconstruction loss and facial consistency loss, we obtain a static Gaussian field that closely adheres to the SMPL-X topology and provides an image-consistent initial human representation for subsequent geometry-aware dynamic adaptation.
- Dynamic Covariance Adaptive Inference(animation phase). This is the core innovation of this study. Specifically, in each frame, we analyze the local topological neighborhood of SMPL-X mesh vertices in real time, calculating the principal stretching direction and magnitude of the surface via Principal Component Analysis (PCA). Subsequently, we explicitly modulate the covariance matrix of the Gaussian primitives, aligning their rotation direction with the surface tangent plane and the axis of stretching, and performing adaptive scaling along the stretching direction. This process requires no additional network training and enables the Gaussian field to maintain continuous surface coverage during animation, thereby reducing motion-induced holes that may affect downstream spatial reasoning and sensing measurements.

Furthermore, since traditional image quality metrics (e.g., PSNR, SSIM) are insensitive to subtle geometric cracks and rely on reference images, we propose the Avatar Surface Integrity Score to quantitatively evaluate the surface integrity of avatars under dynamic deformation. This metric constructs an ideal topological mask through morphological operations and calculates the integrity by combining a density proxy mechanism, thereby providing a reference-free quantitative evaluation standard for the structural continuity of 3D avatars.

### 3.2. Pose-Aware Dynamic Covariance Adaptation Method

The core idea of Dynamic Covariance Adaptation is to anchor each Gaussian primitive to a neighboring SMPL-X vertex, and the primitive’s rotation and scaling attributes are analytically derived from the distribution of the vertex’s local topological neighborhood. In this context, the local topological neighborhood is defined as the set of adjacent vertices associated with all incident triangles shared by the anchor vertex. Let *V* denote the set of all vertices of the SMPL-X mesh. For a given Gaussian primitive *p* whose associated vertex is v∈V, we first define N(v)⊂V as the neighboring vertices directly connected to *v* based on the mesh topology. At each animation frame, the rotation and scaling of *p* are calculated through the following three steps:

1. Tangential plane projection. The *Z*-axis of the Gaussian primitive *p* should be orthogonal to the tangent plane at vertex *v*. We define the normal vector of the tangent plane at *v*, denoted as *Z*, by normalizing the average of the face normals of the *N* triangular facets formed by *v* and the vertices in N(v), as formulated below:
  (1) $$Z=\mathrm{Norm}\left(\frac{\sum_{j=0}^{N-1}\left(u_{j}-v\right)\times \left(u_{(j+1)\%N}-v\right)}{N}\right)$$
  where N=|N(v)|, and $u_{j}\in N\left(v\right)$ (for 0≤j<N) denotes the j-th neighboring vertex, with the vertices ordered counterclockwise around *v*. Here, × denotes the cross product, and Norm(·) represents vector normalization. To endow *p* with a flattened characteristic, ensuring the Gaussian primitive lies flush against the human skin surface, the scaling factor $S_{z}$ of *p* along the *Z* direction is set to a small constant value (in our experiments, $S_{z}=0.001$). We project the relative position vectors of point $u_{j}$ in N(v) onto the tangent plane perpendicular to *Z*. The projection vector $d_{j}$ of neighbor $u_{j}$ is calculated as follows:
  (2) $$d_{j}=\left(u_{j}-v\right)-\left((u_{j}-v)\cdot Z\right)\cdot Z$$
  where (·) denotes the dot product. These projection vectors $D=\{d_{j}|0\leq j<N\}$ represent the local geometric features of the mesh surface around *v* projected onto the tangent plane.
2. Principal Component Analysis. To determine the principal axis of stretching, we first construct the covariance matrix $C\in R^{3\times 3}$ of the neighborhood:
  (3) $$C=\frac{1}{\left|N(v)\right|}\sum_{d_{j}\in D}d_{j}d_{j}^{T}$$
  then perform eigendecomposition on C, obtaining eigenvalues $\lambda_{1}\geq \lambda_{2}\geq \lambda_{3}\approx 0$ and their corresponding eigenvectors $e_{1},e_{2},e_{3}=Z$. $\lambda_{3}\approx 0$ because the neighborhood points are projected onto the tangent plane. $e_{1},e_{2}$ and $\lambda_{1},\lambda_{2}$, respectively, represent the principal distribution directions and spatial variance of N(v) on the tangent plane, which can be used to guide the update of the Gaussian rotation R and scale S.
3. Derivation of dynamic attributes.
  - Rotation alignment. We construct a local orthonormal basis for Gaussian primitive *p* to align with the deformation direction of *p*’s corresponding mesh area. Specifically, the *X*-axis of *p* is aligned to the principal eigenvector $e_{1}$, and its *Y*-axis is derived via the cross product of *Z*-axis and *X*-axis to ensure the right-hand rule of the coordinate system. Since the *Z*-axis of *p* is determined when obtaining the tangent plane, the rotation parameter *R* of *p* is calculated as follows:
    (4) $$R=[e_{1},\;Z\times e_{1},\;Z]$$
  - Adaptive anisotropic scaling. Since the eigenvalues $\lambda_{1}$ and $\lambda_{2}$ represent the deformation amplitudes of the corresponding mesh area of *p* in the directions of $e_{1}$ and $e_{2}$, we define the scaling factors of *p* along the *X*- and *Y*-axes as follows:
    (5) $$S_{x}=\sqrt{\lambda_{1}},\;S_{y}=\sqrt{\lambda_{2}}$$

### 3.3. Avatar Surface Integrity Score (ASIS)

**Figure 3:**
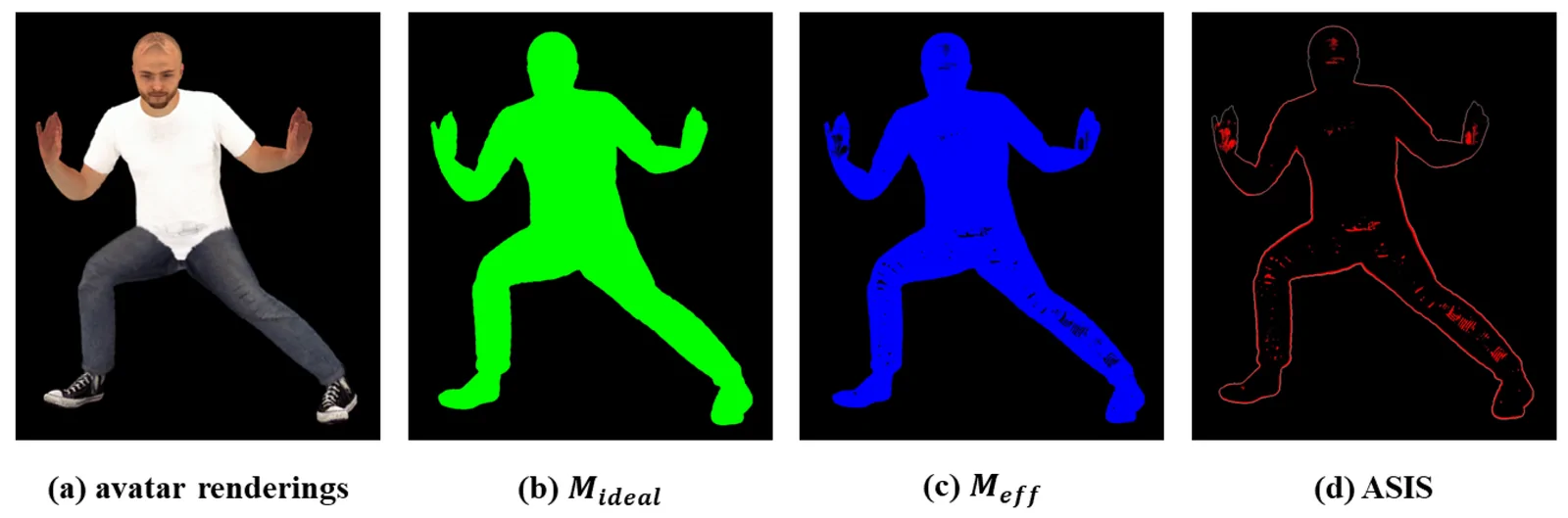
Visualization of intermediate results during the ASIS computation. (**a**) the rendered image of the 3DGS-based avatar; (**b**) the ideal mask representing the human body region ($M_{ideal}$); (**c**) the effective coverage mask derived from Gaussian primitives ($M_{eff}$); and (**d**) the visualization of the ratio between actual coverage and the ideal region, which reflects the surface integrity of the human skin.

By computing the ratio between the effectively rendered density map of the Gaussian field and the ideal body silhouette of the human body, ASIS quantifies the rendering integrity of 3D avatars without the need for reference images. As shown in Figure 2 and Figure 3, The ASIS evaluation process consists of three stages:

1. Ideal morphological mask ($M_{ideal}$). For a given frame *t* during the avatar motion sequence and a specific viewpoint *w*, since all Gaussian primitives possess an opacity parameter α, we first utilize this parameter to render the standard binary alpha mask $A_{raw}^{w,t}$. $M_{ideal}^{w,t}$ is obtained by applying a morphological closing operation to $A_{raw}^{w,t}$, which is implemented as dilation followed by erosion. The “dilation” operation can “heal” subtle unnatural cracks on the manifold surface while preserving large and natural topological gaps (e.g., the gap between the arm and the torso). The erosion operation aims to retrieve the crack-free body silhouette from $A_{raw}^{w,t}$ by restoring its original boundaries. The formula is as follows:
  (6) $$M_{ideal}^{w,t}=A_{raw}^{w,t}\cdot K=\left(A_{raw}^{w,t}⊕K\right)⊖K$$
  where · signifies the morphological closing operator, ⊕ and ⊖ denote the dilation and erosion operators, respectively, and *K* is the structuring element. To suppress jagged boundary artifacts, we employ a 7×7 elliptical structuring element. The morphological operations follow the definitions in [52].
2. Effective density mask ($M_{eff}$). Since each pixel corresponds to multiple Gaussian primitives during the projection onto the 2D image plane, a primitive with an opacity of 1 will fully occlude any subsequent primitives, rendering them invisible. Therefore, we temporarily reset the opacity of all Gaussian primitives to a very small value α (e.g., α=0.05) and render a proxy alpha mask $A_{proxy}^{w,t}$. A pixel in $A_{proxy}$ is classified as “effectively dense” if and only if its cumulative contribution is equivalent to the superposition of at least *N* layers of primitives (*N* is fixed at 2 in our experiments, which is motivated by the entry and exit surfaces encountered by a ray passing through the interior of a closed body). The effective mask is derived as follows:
  (7) $$A_{proxy}^{w,t}=1-{\left(1-\alpha \right)}^{m}$$
  (8) $$M_{eff}^{w,t}=I\left(A_{proxy}^{w,t}\geq \tau_{density}\right)$$
  where *m* represents the number of Gaussian primitives accumulated at that pixel point. The threshold $\tau_{density}$ is calculated based on the cumulative opacity of 2 layers: $\tau_{density}=1-{\left(1-\alpha \right)}^{N}=1-{\left(1-\alpha \right)}^{2}=0.0975$. It is easy to observe that $\tau_{density}$ varies with the value of α, together, α and $\tau_{density}$ define the filtering criterion. The actual factor that determines the effective masking of the avatar area is whether the number of Gaussian primitives *m* corresponding to each pixel point is at least *N*.
3. Avatar Surface Integrity Score (ASIS). To quantitatively evaluate the overall geometric integrity of the human avatar surface, the computation of ASIS requires a comprehensive evaluation of the avatar’s performance across multiple viewpoints throughout the entire motion sequence. This metric first selects a set of orthogonal projection views W={front,right,top} and normalizes the sum by the total area of the ideal human masks across all views to obtain the coverage rate at frame *t*:
  (9) $$H^{t}=\frac{\sum_{w\in W}M_{eff}^{w,t}}{\sum_{w\in W}M_{ideal}^{w,t}}$$
  Subsequently, since the area of surface cracks is relatively small, relying solely on raw pixel coverage is inadequate to distinctly reflect the structural integrity of the avatar. Therefore, we apply a normalized exponential function to scale $H^{t}$ and derive $ASIS^{t}$, as formulated in Equation (10).
  (10) $$ASIS^{t}=\frac{e^{5H^{t}}-1}{e^{5}-1}$$
  Finally, ASIS can be derived from statistical measures (e.g., the mean, median, and variance) of $ASIS^{t}$ estimated over the entire avatar motion sequence. The ASIS statistic used in this study is the mean, which can be calculated using the following formula.
  (11) $$\bar{\mathrm{ASIS}}=\frac{1}{T}\sum_{t=1}^{T}{\mathrm{ASIS}}^{t}$$
  where *T* is the number of evaluated frames in the sequence.

## 4. Results

### 4.1. Experimental Settings

#### 4.1.1. Datasets

To evaluate our method, we conduct experiments on three benchmark datasets:

- X-Humans. X-Humans is a benchmark dataset for expressive full-body human avatar reconstruction, containing high-quality textured human scans, rendered RGB-D images, and corresponding SMPL-X registrations. Its key characteristic is that it supports avatar construction under two data modalities, namely from 3D scan data on the one hand and from RGB-D imagery on the other. In our experiments, we select three subjects, 00028, 00034, and 00087, as evaluation instances since the pretrained weights for the RGB-D setting are publicly available for these identities. Compared with existing methods [13] that rely on RGB-D videos, our method, as well as ExAvatar [4], HAHA [53] and RMAvatar [30], is able to reconstruct avatars using only monocular RGB videos without depth supervision. Following the official protocol of X-Humans, we strictly adopt the dataset’s original training/testing split for all experiments.
- AvatarReX. To further evaluate the robustness of the proposed method in natural scenarios and under expressive motions, we additionally conduct experiments on the AvatarReX dataset. The AvatarReX dataset is constructed from multi-view video captures and contains four samples in total. In our experiments, we evaluate three selected samples and report the average values of all evaluation metrics over these three samples as the final results. To maintain consistency with the monocular reconstruction setting, we select the video sequence from a fixed camera view of each sample as the monocular RGB input.
- People-Snapshot. To broaden the evaluation, we additionally conduct experiments on People-Snapshot, a widely used benchmark for human avatar reconstruction from monocular videos that primarily captures subjects rotating in front of a fixed camera. We reconstruct and evaluate all 12 subject sequences included in our experimental set. These sequences predominantly feature green-screen backgrounds, with subjects wearing solid-colored or simply patterned clothing. We adopt a training–test partitioning strategy based on HAHA [53] and retain the monocular RGB-only reconstruction setting to further evaluate the rendering quality and surface coverage of ElasticGS.

#### 4.1.2. Evaluation Metrics

We employ a comprehensive set of metrics to assess rendering fidelity and geometric integrity:

- Image-level sensing consistency: we report Peak Signal-to-Noise Ratio (PSNR), Structural Similarity Index (SSIM), and Learned Perceptual Image Patch Similarity (LPIPS) [54] to evaluate the photorealism and texture details of the synthesized novel views.
- Surface integrity: we utilize the proposed ASIS to quantify the projected surface integrity and Gaussian coverage compactness. A higher ASIS indicates a more cohesive surface with fewer “cracks.”

#### 4.1.3. Other Experimental Settings

All experiments were conducted on two NVIDIA GeForce RTX 4090 GPUs. To better evaluate the rendering results, the resolution of the rendered images is consistent with that of the dataset images, as follows: the resolution of X-Humans is 800×1200, that of AvatarReX is 1500×2048, and that of People-Snapshot is 1080×1080. The number of Gaussian primitives required to reconstruct the human avatars using different methods varies across different datasets, as shown in Table 1.

### 4.2. Quantitative Evaluation

Table 2 reports quantitative results on the AvatarReX dataset under the monocular video input setting. The results of GaussianAvatar and 3DGS-Avatar are adopted from F3G-Avatar [56], while the results of RMAvatar, ExAvatar and ElasticGS are obtained by reconstructing avatars from single-view RGB videos selected from AvatarReX. For surface integrity evaluation, ASIS is computed for RMAvatar, ExAvatar and ElasticGS under the same reconstruction and proxy-rendering pipeline, enabling a direct comparison of motion-induced surface compactness between the baseline and our method.

Table 2, Table 3, Table 4 and Table 5 show that ElasticGS achieves the highest reported ASIS scores while maintaining competitive rendering performance relative to methods such as RMAvatar and HAHA. Compared with ExAvatar, it consistently improves surface coverage, although these gains are accompanied by reduced rendering fidelity.

On X-Humans (Table 3), ElasticGS achieves the highest ASIS across all three evaluated subjects, increasing the mean score from 0.742 to 0.961 relative to ExAvatar. For subject 00028, ASIS increases from 0.729 to 0.968, while PSNR decreases from 30.201 to 26.860 dB and LPIPS increases from 0.020 to 0.026. These results demonstrate a consistent improvement in surface coverage while explicitly revealing the associated appearance cost.

The same trend is observed on AvatarReX (Table 2): ASIS increases from 0.871 to 0.949, with SSIM remaining at 0.976, while LPIPS increases from 0.034 to 0.043. On People-Snapshot (Table 4), the mean ASIS across 12 sequences increases from 0.874 to 0.952, whereas PSNR decreases from 39.040 to 35.967 dB and LPIPS increases from 0.014 to 0.023. ElasticGS outperforms RMAvatar on all four metrics on this dataset and ranks second in rendering quality behind ExAvatar. The four individual sequences in Table 5 likewise show second-best rendering metrics and the highest reported ASIS.

These consistent findings across three datasets demonstrate that ElasticGS substantially improves avatar surface integrity, as measured by ASIS, while retaining competitive image rendering quality.

### 4.3. Qualitative Evaluation

**Figure 4:**
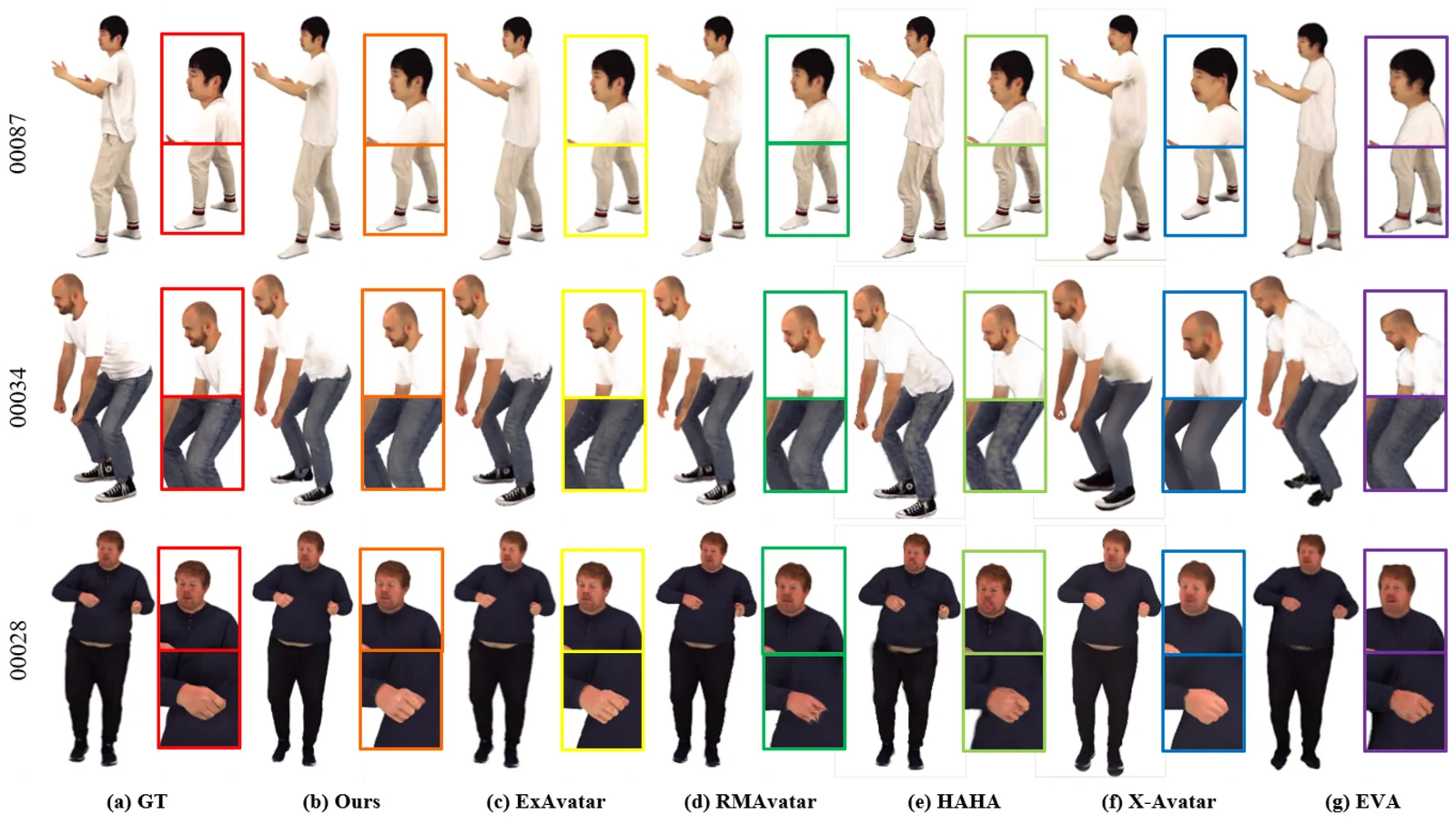
Qualitative comparison of GT, ElasticGS, ExAvatar, RMAvatar, HAHA, X-Avatar, and EVA on the X-Humans dataset.

Figure 4 presents qualitative comparisons on the X-Humans dataset. As shown in Figure 4, HAHA, X-Avatar, and EVA fail to recover sufficient facial details, leg texture and geometry, leading to over-smoothed or incomplete avatar appearances. Compared with these methods, RMAvatar produces clearer body textures and achieves more stable motion reconstruction. However, it still exhibits limitations in fine-detail recovery, including missing regions around the hands and blurred facial details. In comparison, ExAvatar introduces an expressive SMPL-X-based whole-body representation with more detailed hand and facial modeling, allowing it to preserve sharper local texture details. However, ExAvatar still exhibits noticeable surface cracks around the leg regions undergoing pose-driven deformation. This artifact is consistent with the limitation discussed in Section 3.1: Gaussian primitives with fixed or weakly adaptive covariance cannot expand or rotate according to local mesh stretching, and thus fail to cover the enlarged surface area during articulated motion. In contrast, ElasticGS produces a more continuous leg surface. With Pose-Aware Dynamic Covariance Adaptation, Gaussian primitives are dynamically rotated and anisotropically scaled according to local SMPL-X neighborhood deformation, allowing them to adapt to pose-induced stretching and fill potential gaps.

**Figure 5:**
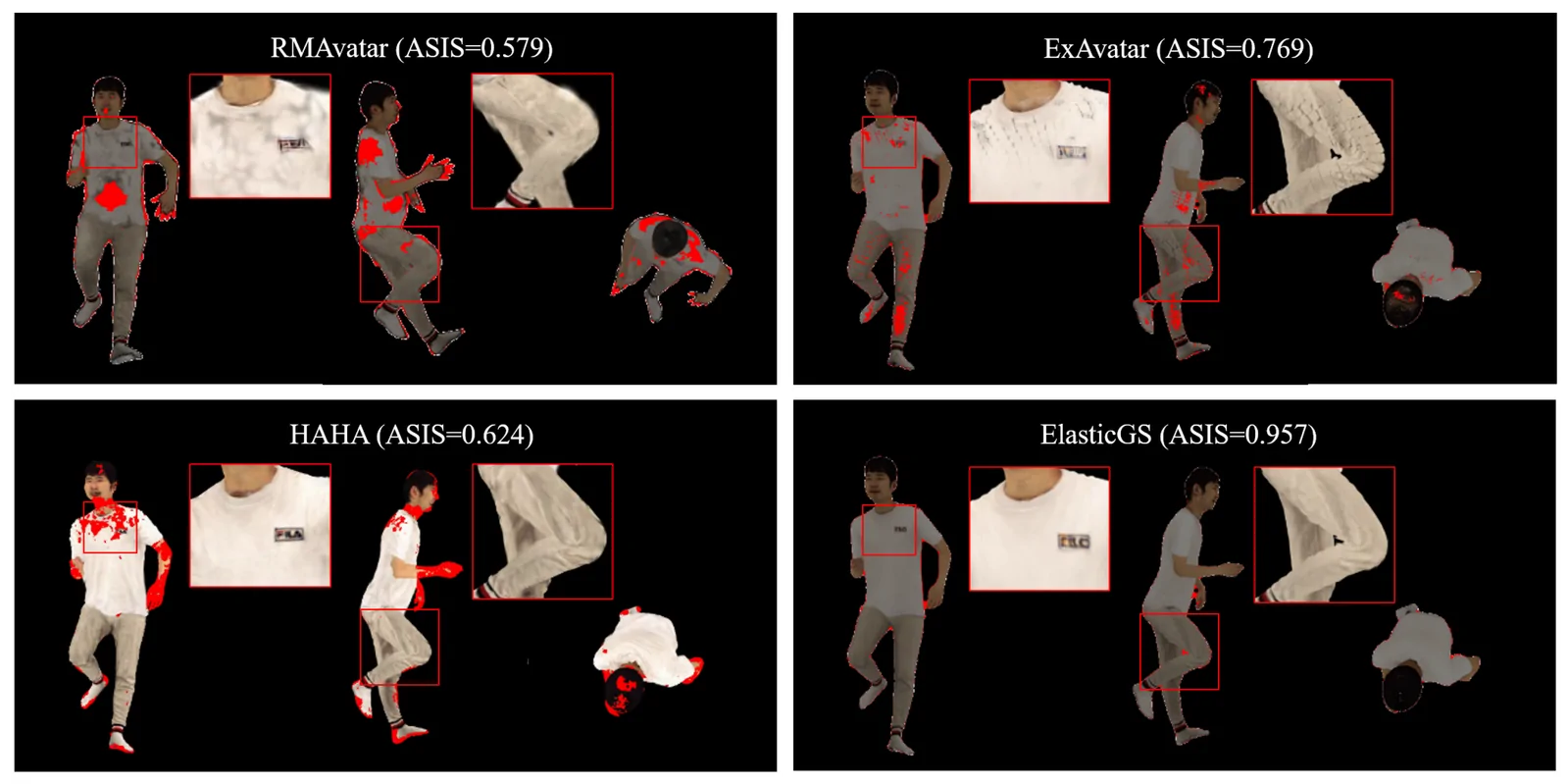
Qualitative comparison among RMAvatar, HAHA, ExAvatar, and our ElasticGS on Subject 00087 from the X-Humans dataset. The figure presents ASIS visualizations across three viewpoints: front, right, and top. The zoom-in patches enclosed in red boxes display the corresponding rendered details in regions with strong articulated deformation.

Figure 5 further compares RMAvatar, HAHA, ExAvatar, and ElasticGS using ASIS heatmaps from the front, right, and top views. RMAvatar exhibits lower-integrity regions around the palm region, abdomen area, and shoulder region. These artifacts occur because the corresponding body parts undergo significant local stretching and geometric deformation during articulation, while the Gaussian primitives cannot sufficiently adjust their spatial covariance to follow the changing surface area. As a result, sparse Gaussian coverage appears as internal holes or low-integrity responses in the ASIS visualization. HAHA improves the overall visual coherence compared with RMAvatar, but still presents several obvious low-density regions in deformation-sensitive areas, especially around the torso, limbs, and articulated junctions, indicating insufficient structural coverage under complex motion. ExAvatar alleviates some of these artifacts, but still shows noticeable low-density regions in strongly stretched body parts. In contrast, ElasticGS presents fewer internal holes and a more compact surface response across all viewpoints. The zoomed-in patches further confirm that our covariance adaptation reduces local discontinuities in deformation-sensitive regions, which is consistent with the ASIS improvements reported in Section 4.2.

Note that red responses along the silhouette boundary do not necessarily indicate real holes. In ASIS, a pixel is considered structurally dense only when its proxy opacity corresponds to at least two accumulated Gaussian layers. However, projected silhouette boundaries are often covered by only one effective layer, so they may be penalized as low-density areas. This explains why the theoretical upper bound of ASIS is 1.0, while the practical maximum is difficult to reach.

### 4.4. Ablation Study

**Figure 6:**
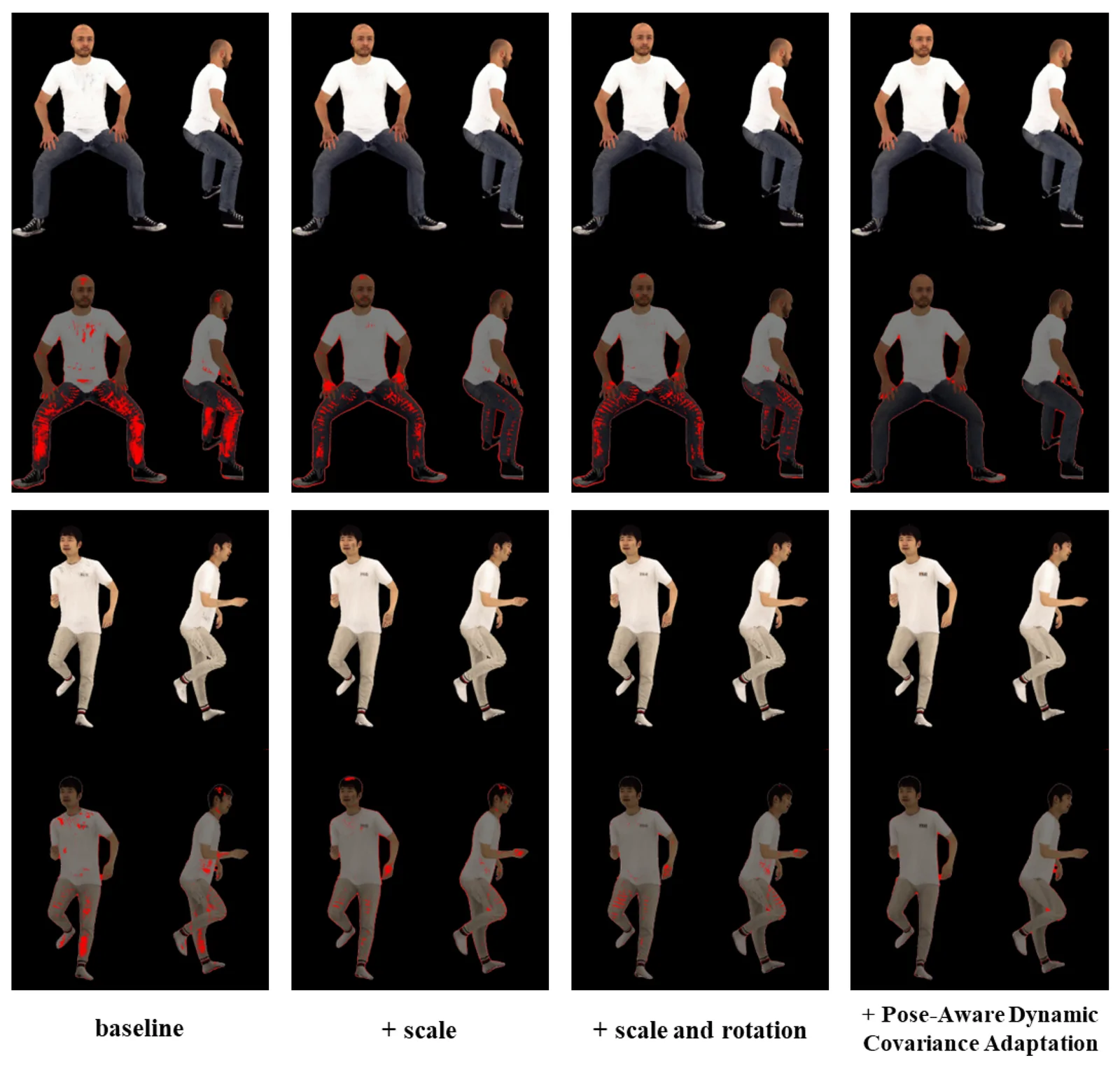
Comparison of different Gaussian deformation strategies.

To validate the contribution of the proposed covariance adaptation, we compare five types of Gaussian deformation strategies in Table 6, with qualitative results for four of these strategies shown in Figure 6. The baseline uses isotropic Gaussian primitives with spherical support and obtains the highest PSNR and optimal LPIPS, but its ASIS is only 0.741, indicating that pixel-level fidelity does not guarantee surface completeness. After introducing anisotropic scaling, ASIS increases to 0.801, showing that enlarging the Gaussian support can partially reduce motion-induced gaps. However, without orientation adjustment, the expanded Gaussians cannot effectively follow the dominant stretching direction of the local surface.

The “+scale and rotation” variant further improves ASIS to 0.896 by aligning the major axis of each anisotropic Gaussian with a heuristic local direction, confirming the importance of directional alignment. Nevertheless, this direction estimated from the farthest vertex pair is sensitive to local topology and may be unstable under complex poses. In contrast, our full Pose-Aware Dynamic Covariance Adaptation achieves an ASIS of 0.961 by using local PCA to estimate the principal stretching direction and dynamically update both Gaussian rotation and anisotropic scale. Although PSNR decreases and LPIPS increases relative to the scale-and-rotation variant, it provides substantially better surface integrity, demonstrating that ElasticGS improves structural completeness of the human surface representation through local deformation-aware covariance modeling rather than simple Gaussian enlargement.

### 4.5. Reliability Analysis of ASIS

**Figure 7:**
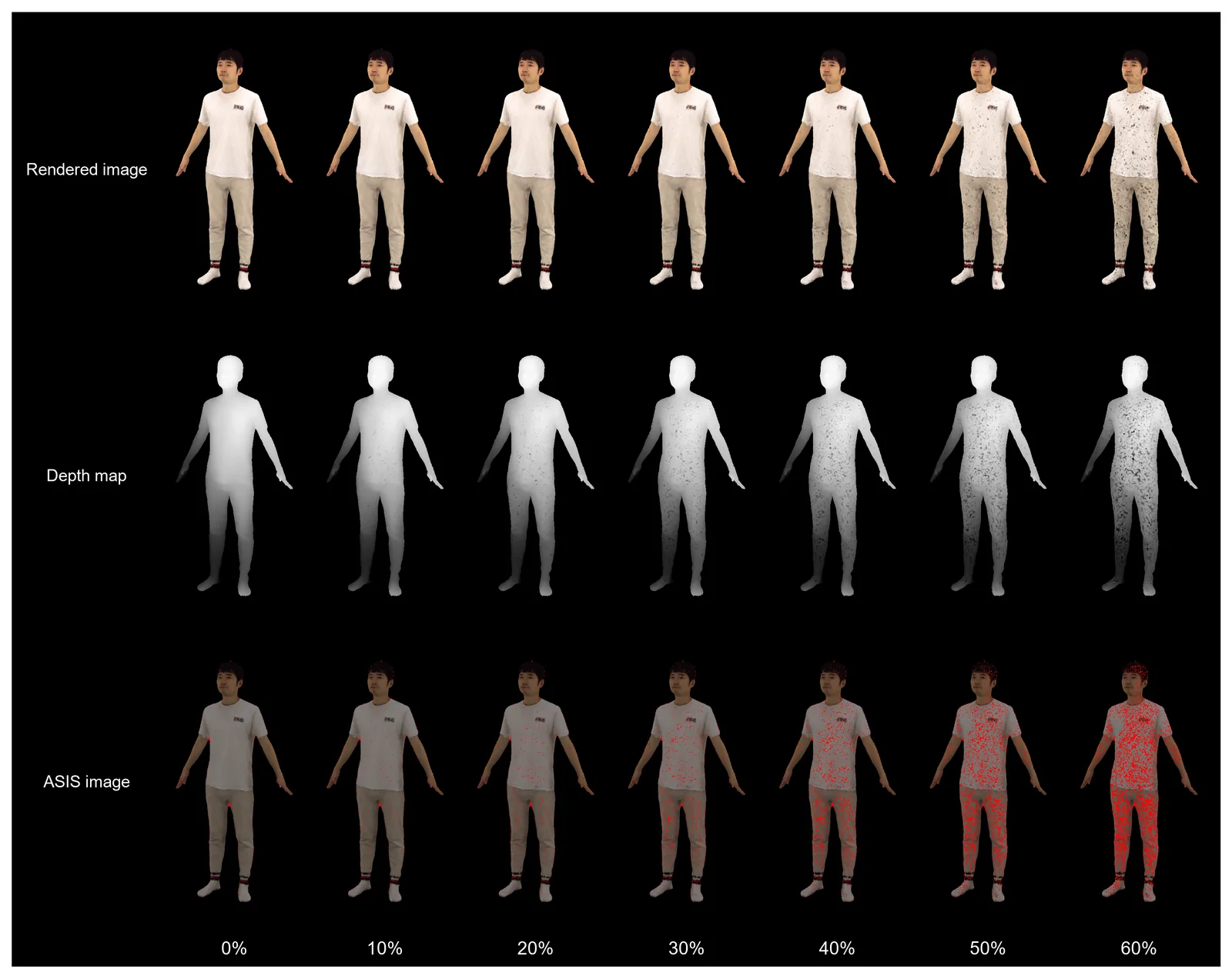
Rendered images, depth maps, and ASIS visualizations of the object 00087 avatar across different Gaussian removal ratios.

As shown in Figure 7, for each object in X-Humans, we take the avatar reconstructed by ElasticGS and randomly remove increasing proportions of Gaussians (from 0% to 60%). For the avatar after each removal, we then evaluate its ASIS, Depth L1 error, Chamfer Distance (CD), and Normal Consistency (NC). Our hypothesis is that, as the removal ratio increases, more holes and gaps will appear on the avatar surface, leading to worse surface integrity. Therefore, as the removal ratio increases, the values of ASIS and NC should decrease, and those of Depth L1 error and CD should increase.

The average surface integrity scores of the three avatars on the X-Humans dataset are shown in Table 7. As the removal ratio increases, ASIS decreases from 0.9614 to 0.2614. Meanwhile, Depth L1 error increased from 12.3721 mm to 38.9389 mm, CD increased from 9.0003 mm to 10.9907 mm, and NC decreased from 0.9069 to 0.9015.

**Figure 8:**
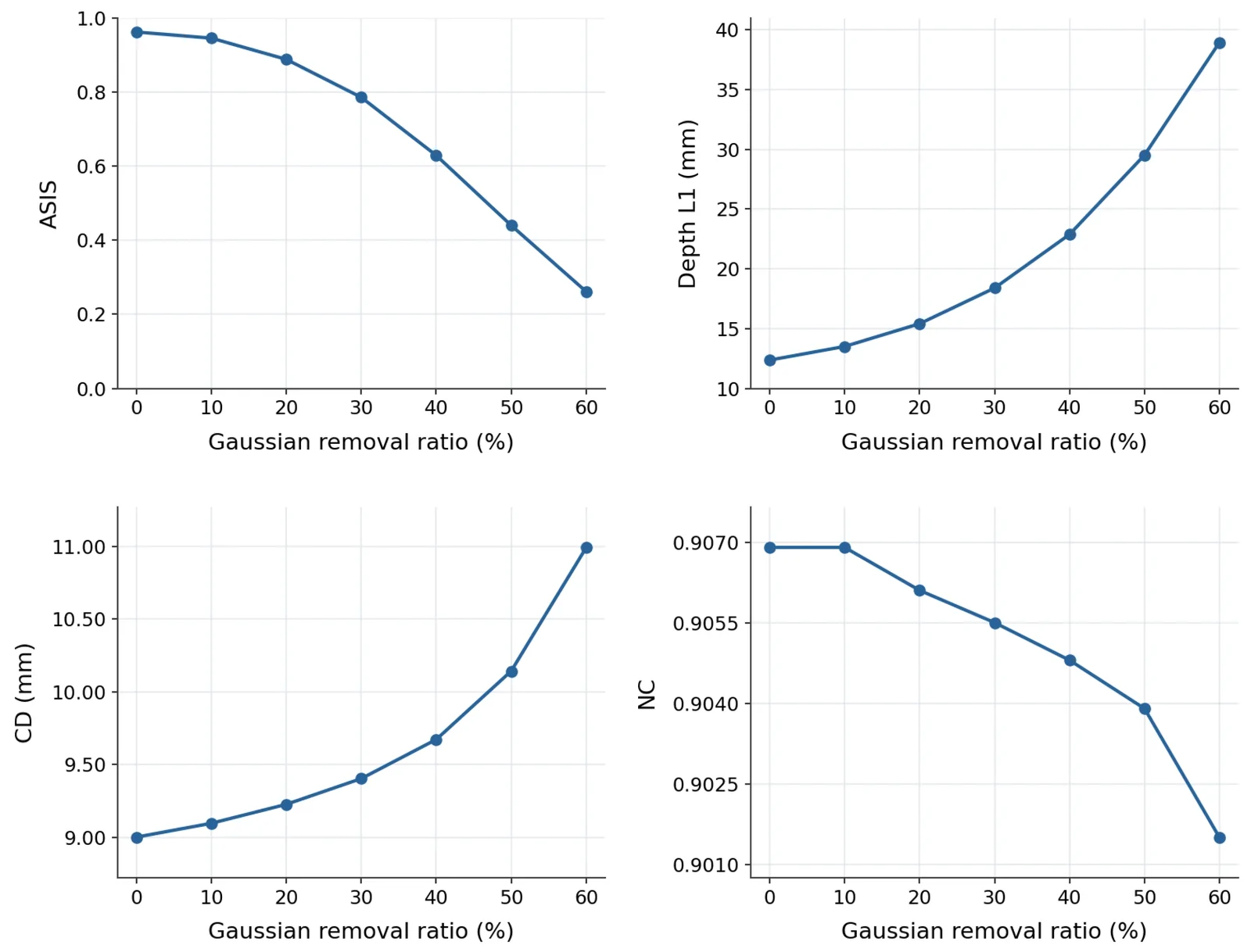
Trends of ASIS, Depth L1, CD, and NC across different Gaussian removal ratios.

As shown in Figure 8, the trends of the four metrics—ASIS, Depth L1, CD, and NC—with increasing Gaussian removal ratio are consistent with our hypothesis. Moreover, ASIS and NC exhibit largely consistent trends, yet ASIS is more sensitive to holes: when the removal ratio reaches 60% and surface holes become highly visible, the value of NC drops by only 0.0054 relative to the 0% removal ratio. Compared with Depth L1 and CD, ASIS shows a closely aligned variation pattern: both remain relatively stable within a 20% removal ratio and deteriorate sharply beyond 30%. These experimental results demonstrate that our proposed ASIS metric, like Depth L1, CD, and NC, is capable of reflecting the degree of surface integrity.

To further examine the ability of ASIS to assess avatar surface completeness, we used four metrics—ASIS, L1 depth error, CD, and NC—to assess the three-dimensional human animation avatars reconstructed by different methods on the X-Humans dataset. As shown in Table 8, ElasticGS achieves the highest ASIS (0.9614) and the lowest depth error (12.3721 mm), indicating that its improved surface coverage is consistent with more accurate rendered depth. ElasticGS and ExAvatar yield identical CD and NC values because these metrics are computed using a proxy mesh defined by Gaussian centers and their connectivity. The ElasticGS method is based on ExAvatar. Its covariance adaptation module automatically scales and rotates the Gaussian primitive without changing the position of the center point of the Gaussian primitive. Therefore, the proxy mesh used in the CD and NC evaluation processes is consistent with that of ExAvatar, and it is unable to capture the improvement in surface integrity resulting from the scaling and rotation of the Gaussian primitive. From the experimental results shown in Table 8, it can be seen that ASIS and Depth L1 are more accurate in evaluating the surface integrity of the avatars reconstructed by different methods compared to CD and NC.

Compared with the geometric metrics like Depth L1, CD, and NC which require ground-truth scans, ASIS requires no ground-truth scans and can directly visualize insufficient surface coverage. This makes it applicable to monocular human reconstruction scenarios where 3D ground truth is unavailable. ASIS focuses on surface completeness, while geometric metrics measure errors in surface position and orientation; the two therefore provide complementary assessments.

### 4.6. Runtime Analysis

Although the proposed covariance adaptation module requires no additional training, it introduces extra computational cost during inference. To quantitatively evaluate this overhead, we conduct a runtime comparison between ExAvatar and ElasticGS on the X-Humans dataset. Specifically, we use the three evaluated subjects, 00028, 00034, and 00087, and report the final runtime results averaged over these subjects.

As shown in Table 9, ElasticGS has almost the same deformation and rendering time as ExAvatar. Specifically, $T_{deform}$ changes from 63.088 ms to 62.976 ms, and $T_{render}$ changes from 3.425 ms to 3.351 ms. This indicates that the proposed method does not noticeably affect the original SMPL-X-driven Gaussian deformation stage or the Gaussian rasterization stage. The main additional cost comes from $T_{adapt}$, which increases from 0.022 ms to 12.406 ms because ElasticGS performs local PCA-based covariance adaptation during inference.

Overall, the total inference time increases from 66.54 ms to 78.73 ms, and the FPS decreases from 15.03 to 12.70. The relative FPS reduction is approximately 15.1%. This confirms that ElasticGS introduces measurable but moderate computational overhead. Considering the substantial improvement in surface integrity, this cost is acceptable for offline reconstruction, interactive visualization, and near-real-time human digital twin applications. Nevertheless, for strict real-time edge deployment, further acceleration is still required.

## 5. Discussion

### 5.1. Limitations of ASIS

Although ASIS provides a practical reference-free proxy for evaluating the surface integrity of animatable 3DGS avatars, it also has several limitations. First, ASIS relies on 2D proxy rendering rather than direct 3D surface measurement. In the reference-free 3DGS avatar setting, directly computing a surface-hole ratio in the 3D domain is not straightforward because Gaussian primitives do not form an explicit watertight mesh surface, and complete ground-truth geometry is usually unavailable during novel-pose animation. Therefore, ASIS estimates surface compactness through projected alpha and density masks.

This projection-based design makes ASIS easy to apply without reference images or complete meshes, but it also introduces view-dependent bias. Surface cracks or density holes that are occluded from the selected viewpoints may not be detected. Conversely, low-density regions near silhouette boundaries may be over-penalized because the projected boundary is often covered by fewer effective Gaussian layers. In addition, the construction of the ideal mask depends on morphological closing. Although this operation can repair small crack-like discontinuities, it may introduce errors under extreme poses, self-contact, or complex clothing. For example, when an arm is close to the torso, morphological closing may incorrectly fill a natural gap; for loose garments such as skirts, the assumed body-like topology may not hold.

Therefore, ASIS should be interpreted as a reference-free proxy metric for surface compactness rather than an absolute measure of 3D geometric accuracy. It is most suitable for comparing different methods under the same viewpoints, rendering settings, morphological parameters, and evaluation protocol. When ground-truth depth maps or complete meshes are available, ASIS should be used together with reference-based geometric metrics and qualitative visualizations.

### 5.2. Trade-Off Between Image-Level Fidelity and Surface Integrity

Although ElasticGS improves surface integrity under large articulated motions, it may reduce image-level fidelity, as reflected by lower PSNR and higher LPIPS. This phenomenon is mainly caused by the geometry-aware expansion and reorientation of Gaussian support. Compared with ExAvatar, which tends to preserve compact Gaussian primitives that are directly optimized for image reconstruction, ElasticGS dynamically adapts the covariance of Gaussian primitives according to local SMPL-X surface deformation. This adaptation increases the spatial support of Gaussians in stretched regions, allowing neighboring primitives to maintain better surface coverage and reducing motion-induced cracks and density holes.

However, the enlarged and anisotropically adjusted Gaussian support may also smooth local high-frequency appearance details or slightly change the projected opacity and color distribution. As a result, pixel-level errors may increase in some regions, even though the reconstructed avatar becomes more geometrically complete. Therefore, the quantitative results should not be interpreted as a uniform improvement over all metrics. Instead, they reflect a trade-off between pixel-level rendering fidelity and geometry-level surface completeness. For sensing-oriented digital human and human digital twin applications, this trade-off can be acceptable because surface continuity is important for reliable visual representation, motion monitoring, and downstream spatial analysis under novel pose driving.

## 6. Conclusions

In this paper, we presented ElasticGS, a geometry-consistent dynamic human reconstruction framework for vision-based 3D sensing and sensor-driven human digital twin applications. The proposed framework addresses the surface discontinuity problem of 3DGS-based animatable human avatars through a training-free pose-aware covariance adaptation strategy. Specifically, the proposed Pose-Aware Dynamic Covariance Adaptation module performs localized PCA on deformed SMPL-X mesh neighborhoods and analytically updates the rotation and anisotropic scale of Gaussian primitives according to local surface stretching. This design enables Gaussian primitives to better follow pose-induced deformation, thereby maintaining more continuous surface coverage and reducing motion-induced cracks and density holes under large articulated motions. In addition, we introduced the Avatar Surface Integrity Score (ASIS), a reference-free proxy-rendering metric for evaluating surface compactness and structural completeness when pixel-aligned ground truth or complete reference geometry is unavailable. Quantitative results on X-Humans, AvatarReX and People-Snapshot demonstrate that ElasticGS substantially improves surface integrity while maintaining competitive image-level sensing consistency. The ablation study further confirms the effectiveness of the proposed covariance adaptation strategy, showing that explicit rotation alignment and anisotropic scaling based on local surface deformation are critical for improving geometric completeness. Overall, this work contributes a geometry-aware Gaussian covariance adaptation framework for robust animatable digital human reconstruction from vision-sensor data.

Despite these advantages, ElasticGS still has several limitations. First, ASIS should be regarded as a reference-free proxy metric rather than an absolute measure of 3D geometric accuracy. Since ASIS relies on 2D proxy rendering and morphological mask construction, it may be affected by silhouette-boundary bias or self-contact. Second, ElasticGS currently focuses on geometry-consistent human avatar reconstruction and pose-driven animation rather than a complete end-to-end digital twin or robotic perception-control system. It has not yet been specifically validated on contact-rich human–object interaction tasks, such as object manipulation, grasping, or catching. In addition, although the current implementation maintains an interactive inference speed, strict real-time edge deployment still requires further acceleration and system-level validation.

Future work will focus on improving the visual quality, robustness, and practical sensing-system integration of ElasticGS. On the reconstruction side, we will investigate adaptive Gaussian densification, view-dependent texture refinement, and deformation-aware appearance compensation to improve fine-grained texture fidelity while preserving surface continuity. For human digital twin and robotics-related applications, we plan to extend ElasticGS by integrating upstream pose tracking, object-aware perception, motion prediction, contact modeling, and collision constraints, so that the reconstructed digital human representation can better support dynamic human–object and human–robot interaction scenarios. We will also evaluate the framework on real-world RGB-D and multi-camera sensing systems for human-centered smart manufacturing monitoring, ergonomic assessment, and safety-aware human-robot collaboration visualization.

## Figures and Tables

**Table 1:** The number of Gaussians required for different methods to reconstruct the avatar.

Methods	X-Humans	AvatarReX	People-Snapshot
EVA [55]	19,993	-	-
GaussianAvatar [43]	-	195,297	-
3DGS-Avatar [8]	-	39,741	-
HAHA [53]	15,365	-	13,635
RMAvatar [30]	96,824	98,541	61,118
ExAvatar [4]	167,390	167,390	167,390
ElasticGS (ours)	167,390	167,390	167,390

**Table 2:** Quantitative results on AvatarReX. Local results for RMAvatar, ExAvatar, and ElasticGS are averaged over the three evaluated subjects using monocular input from a fixed camera per subject. GaussianAvatar and 3DGS-Avatar values are reproduced from F3G-Avatar [56] and marked P. Their subject selection and evaluation implementation have been established as identical to our local protocol. The bolded parts represent the best experimental results. The same applies to the subsequent tables.

Methods	PSNR ↑	SSIM ↑	LPIPS ↓	ASIS ↑
GaussianAvatar [43]	26.950	0.939	0.041	-
3DGS-Avatar [8]	28.784	0.951	0.042	-
RMAvatar [30]	27.173	0.962	**0.032**	0.763
ExAvatar [4]	**31.556**	**0.976**	0.034	0.871
ElasticGS (ours)	30.912	**0.976**	0.043	**0.949**

**Table 3:** Quantitative comparisons of 3D human avatars on the test set of X-Humans. ↑/↓ indicate that higher/lower values are better; “-” denotes results not reported.

Methods	00028				00034				00087			
	PSNR ↑	SSIM ↑	LPIPS ↓	ASIS ↑	PSNR ↑	SSIM ↑	LPIPS ↓	ASIS ↑	PSNR ↑	SSIM ↑	LPIPS ↓	ASIS ↑
EVA [55]	**31.840**	0.960	0.055	-	25.572	0.947	0.045	-	27.507	0.961	0.028	-
X-Avatar [13]	28.573	0.976	0.026	-	28.052	**0.965**	0.035	-	30.894	0.970	0.030	-
HAHA [53]	25.425	0.961	0.036	0.881	24.560	0.941	0.043	0.724	26.977	0.934	0.045	0.624
RMAvatar [30]	27.561	0.976	0.025	0.797	26.624	0.958	0.033	0.623	30.336	0.966	0.028	0.579
ExAvatar [4]	30.201	**0.982**	**0.020**	0.729	**28.137**	**0.965**	**0.026**	0.727	**31.772**	**0.971**	**0.027**	0.769
ElasticGS (ours)	26.860	0.976	0.026	**0.968**	27.042	0.961	0.035	**0.960**	30.894	0.968	0.030	**0.957**

**Table 4:** Quantitative results on People-Snapshot. Results are averaged over the 12 evaluated sequences. All methods were evaluated locally using an NVIDIA GeForce RTX 4090 GPU.

Methods	PSNR ↑	SSIM ↑	LPIPS ↓	ASIS ↑
RMAvatar [30]	26.233	0.968	0.035	0.815
ExAvatar [4]	**39.040**	**0.987**	**0.014**	0.874
ElasticGS (ours)	35.967	0.982	0.023	**0.952**

**Table 5:** Quantitative results for four selected sequences from People-Snapshot.

Methods	Male-3-Casual				Male-4-Casual				Female-3-Casual				Female-4-Casual			
	PSNR ↑	SSIM ↑	LPIPS ↓	ASIS ↑	PSNR ↑	SSIM ↑	LPIPS ↓	ASIS ↑	PSNR ↑	SSIM ↑	LPIPS ↓	ASIS ↑	PSNR ↑	SSIM ↑	LPIPS ↓	ASIS ↑
HAHA [53]	31.462	0.962	0.028	-	27.083	0.951	0.043	-	32.531	0.963	0.040	-	31.152	0.959	0.028	-
RMAvatar [30]	27.151	0.966	0.030	0.782	26.914	0.971	0.034	0.885	25.633	0.968	0.033	0.883	26.814	0.967	0.029	0.870
ExAvatar [4]	38.572	0.984	0.015	0.853	39.210	0.989	0.014	0.908	40.095	0.985	0.016	0.904	39.152	0.985	0.012	0.909
ElasticGS (ours)	36.451	0.979	0.021	0.953	33.872	0.982	0.028	0.951	37.101	0.980	0.027	0.957	36.138	0.979	0.019	0.953

**Table 6:** Quantitative comparisons of different Gaussian deformation strategies on the X-Humans dataset. “Global scaling” uniformly multiplies the scale of each trained ExAvatar Gaussian along all three axes by a constant c, while keeping all other parameters unchanged. “+scale” indicates the introduction of anisotropic scaling during the training process to convert isotropic Gaussians into ellipsoidal primitives, while “+scale and rotation” additionally aligns the major axis of each anisotropic Gaussian with the direction connecting the farthest pair of vertices in its local neighborhood. “+Pose-Aware Dynamic Covariance Adaptation” is applied during the inference stage.

Methods	PSNR ↑	SSIM ↑	LPIPS ↓	ASIS ↑
ExAvatar (baseline)	30.037	0.973	0.025	0.741
+Global scaling (c = 1.2)	28.733	0.968	0.033	0.908
+Global scaling (c = 1.4)	28.208	0.967	0.036	0.949
+Global scaling (c = 1.6)	27.470	0.965	0.040	0.960
+Global scaling (c = 1.8)	26.686	0.962	0.044	0.966
+scale	28.952	0.968	0.030	0.801
+scale and rotation	28.769	0.968	0.027	0.896
+Pose-Aware Dynamic Covariance Adaptation	28.279	0.969	0.031	0.961

**Table 7:** Quantitative evaluation of avatar surface integrity across different metrics on the X-Humans dataset. Depth L1 error (Depth_L1) and Chamfer Distance (CD) are reported in millimeters. “↑” indicates that a higher value is better for this metric, and “ ↓” indicates that a lower value is better.

Gaussians Removal Ratio	ASIS ↑	Depth_L1 ↓	CD ↓	NC ↑
0%	0.9614	12.3721	9.0003	0.9069
10%	0.9448	13.5128	9.0953	0.9069
20%	0.8876	15.4163	9.2257	0.9061
30%	0.7852	18.3986	9.4027	0.9055
40%	0.6286	22.8829	9.6717	0.9048
50%	0.4399	29.5174	10.1420	0.9039
60%	0.2614	38.9389	10.9907	0.9015

**Table 8:** Quantitative evaluation of avatar surface integrity across different methods on the X-Humans dataset.

Methods	ASIS ↑	Depth_L1 ↓	CD ↓	NC ↑
RMAvatar [30]	0.6663	25.9872	32.4595	0.6766
ExAvatar [4]	0.7416	17.5276	9.0003	0.9069
ElasticGS (ours)	0.9614	12.3721	9.0003	0.9069

**Table 9:** Runtime comparison between ExAvatar and ElasticGS on the X-Humans dataset. The reported values are averaged over subjects 00028, 00034, and 00087. The inference process is decomposed into three components: $T_{deform}$, $T_{adapt}$, and $T_{render}$. $T_{deform}$ denotes the time required to generate Gaussian deformation and attributes from SMPL-X parameters, $T_{adapt}$ denotes the time consumed by the proposed covariance adaptation module, and $T_{render}$ denotes the time for Gaussian rasterization rendering.

Methods	$T_{\mathrm{deform}}$ (ms)	$T_{\mathrm{adapt}}$ (ms)	$T_{\mathrm{render}}$ (ms)	$T_{\mathrm{total}}$ (ms)	FPS
ExAvatar [4]	63.09	0.02	3.43	66.54	15.03
ElasticGS (ours)	62.98	12.41	3.35	78.73	12.70

## Data Availability

Additional experimental results and source code are available at https://github.com/jingyuxiaonan/ElasticGS (accessed on 30 September 2026).
